# Seasonal succession of endophyte and the association with active ingredients in *Rheum palmatum*

**DOI:** 10.1128/spectrum.01184-24

**Published:** 2024-09-24

**Authors:** Ni Li, YiFan Mao, YaLi Huang, LingXuan Zhang, Lu Hou, XiaoJun Liu, YaRong Du, DaWei Chen, Kun Sun

**Affiliations:** 1 College of Life Sciences, Northwest Normal University, Lanzhou, Gansu, China; 2 Key Laboratory of Strategic Mineral Resources of the Upper Yellow River, Ministry of Natural Resources, Lanzhou, Gansu, China; 3 Key Laboratory of Space Radiobiology of Gansu Province & CAS Key Laboratory of Heavy Ion Radiation Biology and Medicine, Institute of Modern Physics, Chinese Academy of Sciences, Lanzhou, Gansu, China; Agroscope, Nyon, Switzerland

**Keywords:** endophyte, seasonal succession, *Rheum palmatum*, correlation analysis, microbial network

## Abstract

The endophyte is closely related to medicinal plant growth and development, stress resistance, and active ingredients’ accumulation. However, a seasonal succession of endophytes and the association with active ingredients is still unclear. In this study, we used high-throughput sequencing methods to compare the endophyte diversity of *Rheum palmatum* under different seasons and analyze the association between endophytes and five active ingredients. The results show that the diversity of endophytic fungi increased and then decreased, while bacterial diversity increased with the change of season. Community composition showed that the dominant genera of endophytic fungi were different under the different seasons, while the dominant genera of endophytic bacteria were *Delftia*. Analysis of co-occurrence network maps showed that the connectivity and complexity of endophytic fungi and bacterial networks decreased with the change of season. Spearman analysis indicated that the active ingredients of *R. palmatum* were significantly positive correlation with genera of endophytic fungi (*Chalara*). FUNGuild and PICRUSt predictive analysis indicated that the function of endophytic fungi and bacteria, respectively, were symbiotroph and metabolism, and relative abundances were different under the different seasons. Our results help elucidate the mechanism of medicinal plant–endophyte interaction.

**IMPORTANCE:**

Through the investigation of the seasonal succession of endophytes and the association with active ingredients in *Rheum palmatum*, we found that the diversity and composition of endophytes in *R. palmatum* exhibited seasonal dynamics, and the active ingredients of *R. palmatum* showed a significantly positive correlation with the genus of endophytic fungi (*Chalara*). Our results may lay a foundation for understanding the interaction mechanism of endophyte and medicinal plant, and can also provide a theoretical basis for sustainable production of medicinal plants.

## INTRODUCTION

Endophytes inhabited the tissues of almost all plants without causing any disease symptoms (1). Endophytes play an important role in promoting plant growth and development, and stress tolerance, especially stimulating active ingredients of medicinal plants (2–5). Tao et al. isolated four strains of endophytic bacteria from *Pairs polyphylla*, which significantly increased the host’s biomass (6). Gao et al. reported that endophytic *Paenibacillus polymyxa* can promote *P. ginseng* growth, improve ginsenoside content, and decrease plant disease (7). Eid et al. found that endophytes also help to promote host plant growth, increase the absorption of nutrients, reduce the debilitating effects of diseases, and improve host resistance against environmental stresses via active ingredients’ accumulation (8). Therefore, analysis of the diversity and composition of endophytes could provide valuable resources for regulating the synthesis and accumulation of active ingredients in medicinal plants.

Many reports that the synthesis and accumulation of active ingredients of medicinal plants exist in seasonal dynamics (9, 10). Silva et al. found that the active ingredients of *Vitex negundo* are β-caryophyllene and cineole, which have the highest content in November and January, respectively (11). Wang et al. found that the best harvest season for the three *Sedum* medicinal species should be the full-bloom period between the end of April and the beginning of May (12). Liao et al. concluded that the shoots of *Oxalis corymbosa* may have good oxidation resistance during winter (13). Meanwhile, many previous works highlighted that seasonality is a key factor driving the environment, composition, and diversity of microorganisms (14–16). Such as temperature, light, and rainfall, all show obvious synergistic changes with the seasons, and directly or indirectly affect the structure, diversity, and function of microbial communities (17). However, studies on the seasonal succession of endophytes are still limited in medicinal plants.

*Rheum palmatum* is a perennial herb of the genus *Rheum* in the Polygonaceae family, which is a famous Traditional Chinese Medicines (TCM), mainly using dried roots as medicine. The active ingredients were mainly anthraquinones, including aloe-emodin, rhein, emodin, chrysophanol, and physcion (17). It can treat many diseases such as constipation, jaundice, and ulcers because of its excellent bioactivities (18). In previous work, we found that there were differences in endophyte diversity and community composition in different regions, ages, and tissue types of *R. palmatum* (19, 20) and that the active ingredients were positively correlated with the diversity of endophytic fungi (19). Meanwhile, we have isolated endophytic *Trichoderma citrinoviride* HT-1 from *R. palmatum* root, which can promote the growth, development, and accumulation of active ingredients (21). However, the seasonal succession of endophytes and the association with active ingredients of *R. palmatum* are still unknown. Therefore, we used amplicon sequencing to compare the endophyte diversity of *R. palmatum* under the different seasons and to analyze the association between endophyte and five active ingredients. These results may lay a foundation for understanding the interaction mechanism of endophyte and medicinal plant, and can also provide a theoretical basis for sustainable production of medicinal plants.

## MATERIALS AND METHODS

### Experimental materials

Three-year-old roots of *R. palmatum* were collected from Xuanshui, Lixian county, Gansu Province, China (104°77′89″, 32°85′26″,736 m) in different seasons (spring, Ar; summer, Br; and autumn, Cr). Three biological replicates were selected for uniformity based on size and weight. 0.5 g of root (principal root) of *R. palmatum* was collected from each season as a sample, which kept a 10–15 cm distance from the stem base. The samples were separated and washed with running tap water and then rinsed thrice with distilled water. To sterilize the surface of the plant parts, the root samples were successively immersed in 70% ethanol for 5 min, 2.5% sodium hypochlorite for 1–2 min, and 70% ethanol for 1 min, and then rinsed five times with sterile Millipore water. The last portion of the washing water was inoculated in potato dextrose agar (PDA) at 28°C for 10 days and nutrient agar (NA) at 37°C for 3 days to validate sterilization efficiency. All samples were stored at −80°C until DNA extraction.

### DNA extraction, polymerase chain reaction (PCR) amplification, and sequence processing

The total genomic DNA was extracted from all samples by using the MOBIO Power-Soil Kit (MOBIO Laboratories, Inc., Carlsbad, CA, USA), according to the manufacturer’s instructions. The DNA extracts were analyzed for their concentration using a NanoDrop spectrophotometer (Thermo Fisher Scientific, Model 2000, Waltham, MA, USA) and stored at −20°C for further PCR amplification. The PCR assays were performed in 20 µL mixture containing 4 µL of 5 × Fast Pfu buffer, 2 µL of 2.5 mM dNTPs, 0.8 µL of each primer 5 µM, 0.4 µL of FastPfu Polymerase, 10 ng of template DNA, and ddH_2_O. Bacterial 16S gene was amplified with primers 799F (5′-AACMGGATTAGATACCCKG-3′) and 1193R (5′-ACGTCATCCCCACCTTCC-3′). Amplification was performed under the following conditions: initial denaturation at 95°C for 3 min, 30 cycles at 95°C for 30 s, 52°C for 30 s, and 72°C for 45 s, and a final extension at 72°C for 5 min. The fungal ITS genes were amplified using the primers fITS7 (5′-GTGARTCATCGAATCTTTG-3′) and ITS4 (5′-TCCTCCGCTTATTGATATGC-3′). The PCR reactions were conducted using the following program: 3 min of denaturation at 95°C, 35 cycles of 30 s at 95°C, 30 s for annealing at 55°C, and 45 s for elongation at 72°C, and a final extension at 72°C for 10 min. The PCR products were analyzed by agarose gel electrophoresis. For each sample, three successful PCR products were pooled and purified using EasyPureTM PCR Clean up/Gel Extraction Kit (Axygen Biosciences, Union City, CA, USA) according to the manufacturer’s instructions. Purified amplicons were sequenced on an Illumina NovaSeq platform for paired ends according to the standard protocols (22).

### Active ingredients of *R. palmatum* quantitative analysis

Standard aloe-emodi, rhein, emodin, chrysophanol, and physcion were obtained from the Shanghai R&D Center for Standardization of TCM. High-performance liquid chromatography (HPLC)-ultrapure water, analytical-grade methanol, NaHCO_3_, and phosphoric acid were purchased from Sangon Biotech, Ltd. (Shanghai, China).

According to the Chinese Pharmacopoeia (Edition 2015) (23), the dried root of each season (spring, summer, and autumn) was pulverized and sieved through a 300 µm mesh. A total of 1.5 g of powder of each sample was precisely weighed, added to 10 mL 0.1% NaHCO_3_ aqueous solution, and treated with ultrasound (30–40°C, 700 W) for 20 min. Then, 40 mL methanol was added for ultrasonic treatment for 50 min. Filtrate was obtained by filtration of 0.22 µm Millipore filter unit, and 10 µL of sample solution was injected into HPLC for determination.

According to the method of Chen et al. (24), the samples were analyzed by HPLC (Waters) using C18 (4.6 × 250 mm, 5.0 µm, Waters E2695, Milford, MA, USA) at 30°C, and the content of metabolites was determined: The mobile phase was methanol −0.1% phosphoric acid (80:20). The flow rate was 1 mL·min^−1^. The detection wavelength was 254 nm.

### Analysis of amplicon sequencing data

The data were analyzed by utilizing the QIIME pipeline, as previously performed in (25). Fungal and bacterial sequences were trimmed and assigned to each sample based on their barcodes. Operational taxonomic units (OTUs) at the species level by searching all sequences against the Silva bacterial 16S database (26). OTUs were classified at the species level by searching against the UNITE fungal database (27). Sequences were binned into OTUs at a 97% similarity level by using USEARCH software (http://drive5.com/uparse/, accessed on November 14, 2020) (26).

### Statistical analysis

With QIIME software (Version 1.9.1), blast method (25) (http://qiime.org/scripts/assign_taxonomy.html) in the UNITE (version) database (https://unite.ut.ee/. PHP) (28) annotated the OTUs sequences of samples and analyzed the community composition of endophytes of *R. palmatum* under different seasons. QIIME (Version 1.9.1) was used to analyze the community diversity index of endophytes of *R. palmatum* in different seasons, including good coverage, Chao 1, Shannon, and other indicators (19). The stacked bar chart of species composition was used to represent the composition of various species. The visual distribution of the composition of each sample in phylum and genus classification levels was realized through statistical analysis of the feature table after the removal of singleton, and the analysis results were presented in the bar chart. Nonmetric multidimensional permutations analysis (NMDS) was used to analyze the discrepancies between the samples at the level of OTUs based on the Bray–Curtis distance. The correlation and visual analysis were carried out using packages such as vegan, ggplot2, psych, and WGCNA in R 3.6.3. Metabolic and ecologically relevant functions were annotated by PICRUSt for the 16S rDNA OTU and FUNGuild v1.0 for the ITS OUT (29).

### Co-occurrence network analysis

OTUs with 50% samples were screened to construct the correlation matrix. The coexisting network was constructed with correlation coefficient *R* > 0.6 or <−0.6 and significance *P* < 0.05 as the threshold. The core flora was defined according to the degree, intermediate centrality, and compact centrality. Force Atlas2 layout algorithm of GePhi 0.9.2 (https://gephi.org/) was used to visualize the community network structure of endophytes in *R. palmatum* in different seasons based on a species-level matrix.

## RESULTS

### Surface-sterilization efficiency

The results showed that no colonies were found in the PDA and NA medium after a period of cultivation. It indicated that surface sterilization was effective and ready for subsequent experiments.

### Analysis of sequencing data and alpha diversity

**Fig 1:**
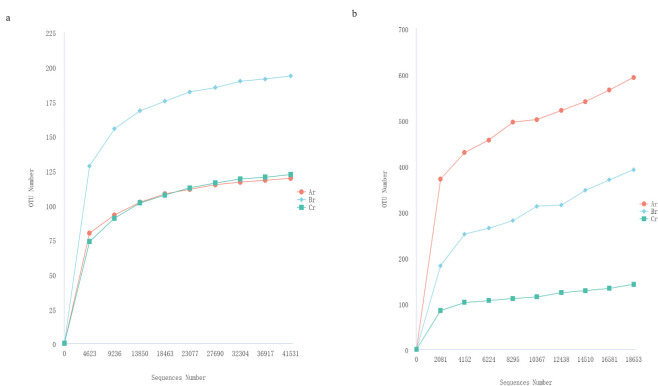
Rarefaction curves based on pyrosequencing of endophytic fungi (a) and bacteria (b) of root for each sample.

The results of the rarefaction curve can reveal the changes in species diversity and richness of samples with sequencing amount (28, 30). As shown in Fig. 1, the rarefaction curves of the samples based on the number of observed species tended to be stable with the increase in the amount of sequencing effort, which proved that sequencing data were gradually becoming reasonable.

A total of 299,252 and 293,298 effective tags were obtained for the fungal and bacterial samples, respectively, after filtering and removing chimeric particles, with the library coverage of the samples being higher than 0.988, which indicates that the sequencing data confidently reflected the structure of the endophytic fungi and bacteria community of the samples. Alpha diversity indices (Chao1 and Shannon’s diversity index) presented differences among all samples of *R. palmatum*. Chao1 indicated a fungal and bacterial community richness trend of Br >Cr > Ar and Ar >Br > Cr, respectively. Shannon’s indicated the fungal and bacterial community richness trend of Br >Ar > Cr and Ar >Br > Cr (Table 1).

**TABLE 1:** Community diversity of endophytes under different seasons samples

Sample	Endophytic fungi				Endophytic bacteria			
	Effective tags	Shannon	Chao1	Good-coverage	Effective tags	Shannon	Chao1	Good-coverage
Ar	100,462	3.19 ± 0.09b	121.03 ± 3.57b	0.999	93,107	2.44 ± 0.78 a	588.31 ± 59.89 a	0.994
Br	99,537	3.51 ± 0.07 a	193.33 ± 4.22 a	0.999	103,018	1.23 ± 0.27b	402.16 ± 56.76b	0.995
Cr	99,253	2.91 ± 0.057 c	124.07 ± 7.03b	0.999	97,173	0.83 ± 0.14 c	147.6 ± 42.67 c	0.998

**Fig 2:**
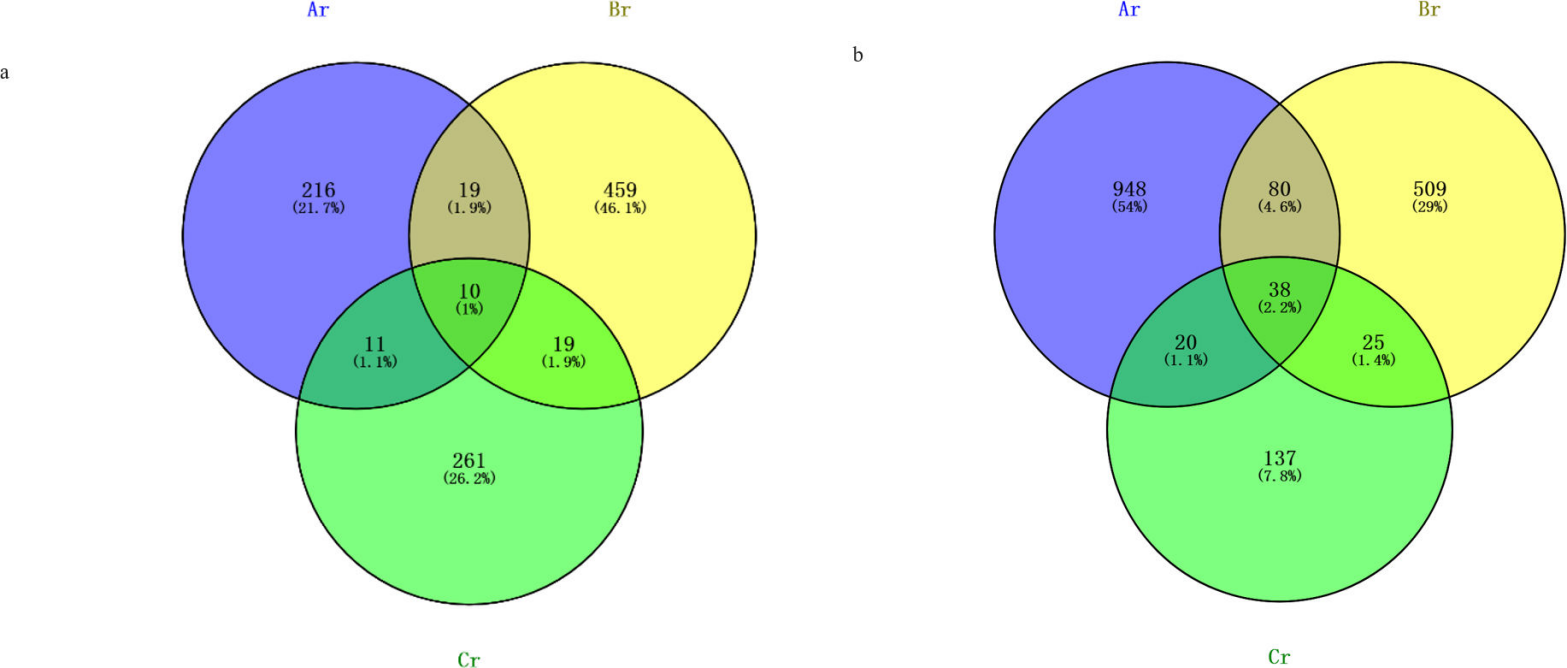
Venn diagram showing the fungal OTUs of different seasons samples (a) and bacterial OTUs of different seasons samples (b).

In all libraries, 10 fungal OTUs and 38 bacterial OTUs were shared under different seasons samples. The numbers of fungal OTUs that occurred only in spring roots, summer roots, and autumn roots samples were 216, 459, and 261, respectively, while the numbers of bacterial OTUs that occurred only in spring roots, summer roots, and autumn roots samples were 948, 509, and 137, respectively (Fig. 2).

### Community composition

**Fig 3:**
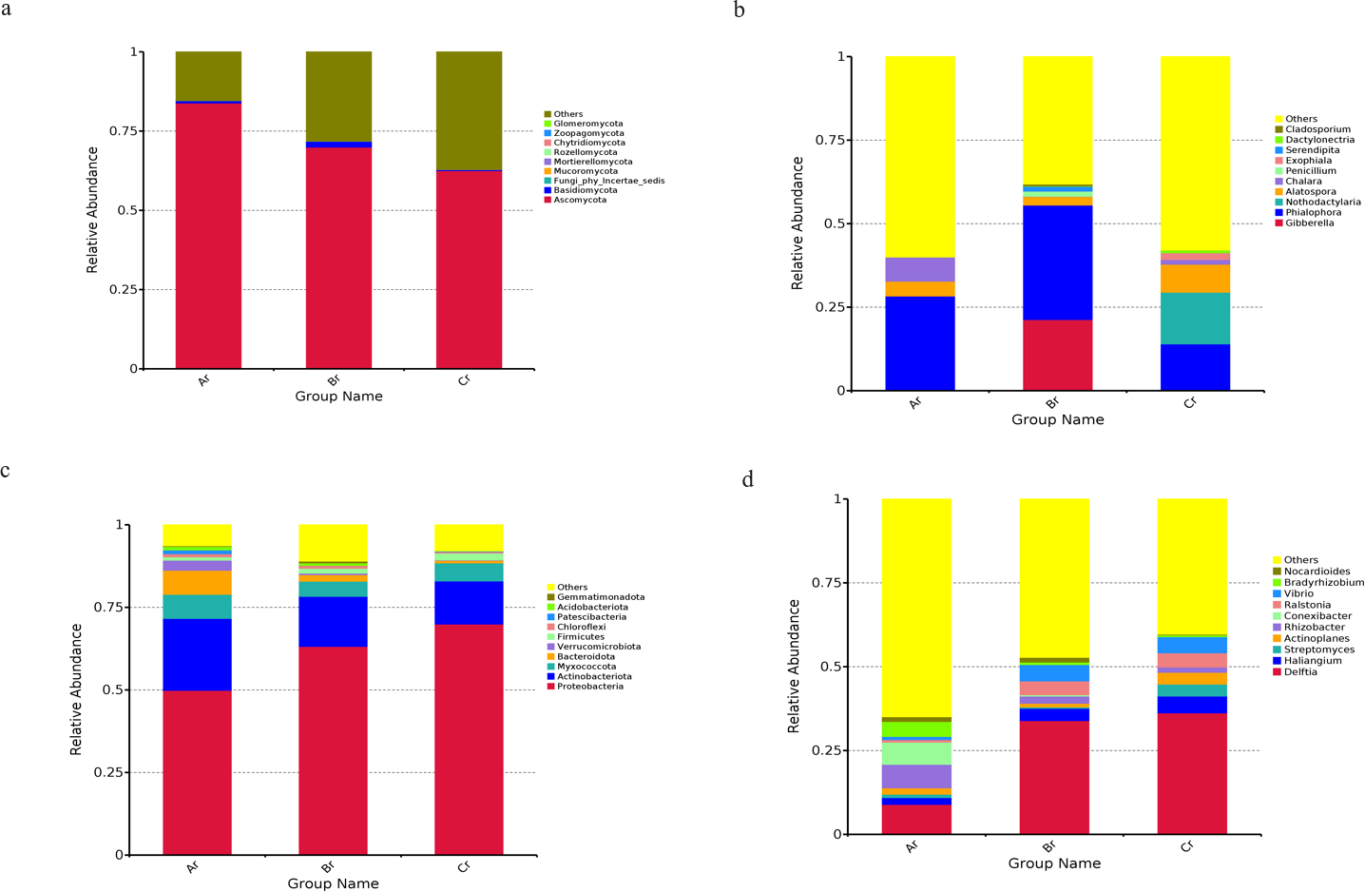
Relative abundances of the endophytic fungi at the phylum level (a), endophytic fungi at the genus level (b), endophytic bacteria at the phylum level (c), and endophytic bacteria at the genus level (d) for each sample. Relative abundances are based on the proportional frequencies of the DNA sequences that could be classified. “Other” represents the total relative abundance outside the top 10 maximum relative abundance levels.

The fungal OTUs were assigned into 10 phyla and 190 genera. The dominant fungal phylum across all of the samples was Ascomycota, with relative abundances ranging from 62.54% to 83.85% (Fig. 3a). At the genus level, *Phialophora* was the dominant genus in the sample of Ar and Br (28.29% and 34.24%, respectively), *Nothodactylaria* was the dominant genus in the sample of Cr (15.51%). (Fig. 3b).

The bacterial OTUs were assigned to 30 phyla and 307 genera. The dominant bacterial phylum across all of the samples was Proteobacteria, with relative abundances ranging from 49.89% to 69.94% (Fig. 3c). At the genus level, *Delftia* was the dominant genus in all samples, with relative abundances ranging from 8.94% to 36.20%. (Fig. 3d).

Each point in the NMDS plot represents a sample, and different colored points indicate different samples (groups). Since NMDS uses rank ordering, it can be approximated that the closer (far) the distance between two points, the smaller (greater) the difference in the microbial communities in the two samples.

**Fig 4:**
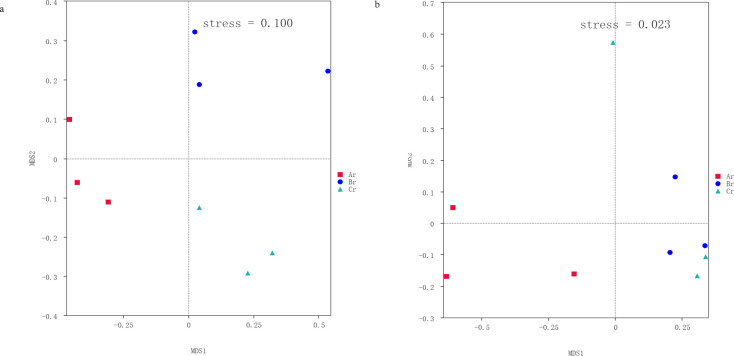
NMDS results of fungal (a) and bacterial (b) community compositions. The digital number represented three biological replicates for each sample.

NMDS analysis showed that the fungal and bacterial community composition was generally separated when comparing roots from different seasons (Fig. 4). This implied that the community structure of fungi and bacteria exists difference under different seasons.

### Co-occurrence network analysis

To explore whether changes in microbial community assemblies were accompanied by changes in microbial interactions, we performed a co-occurrence network analysis and estimated the topological properties to uncover the complexity of potential associations and connections between endophytic microorganisms of the root under different seasons.

**Fig 5:**
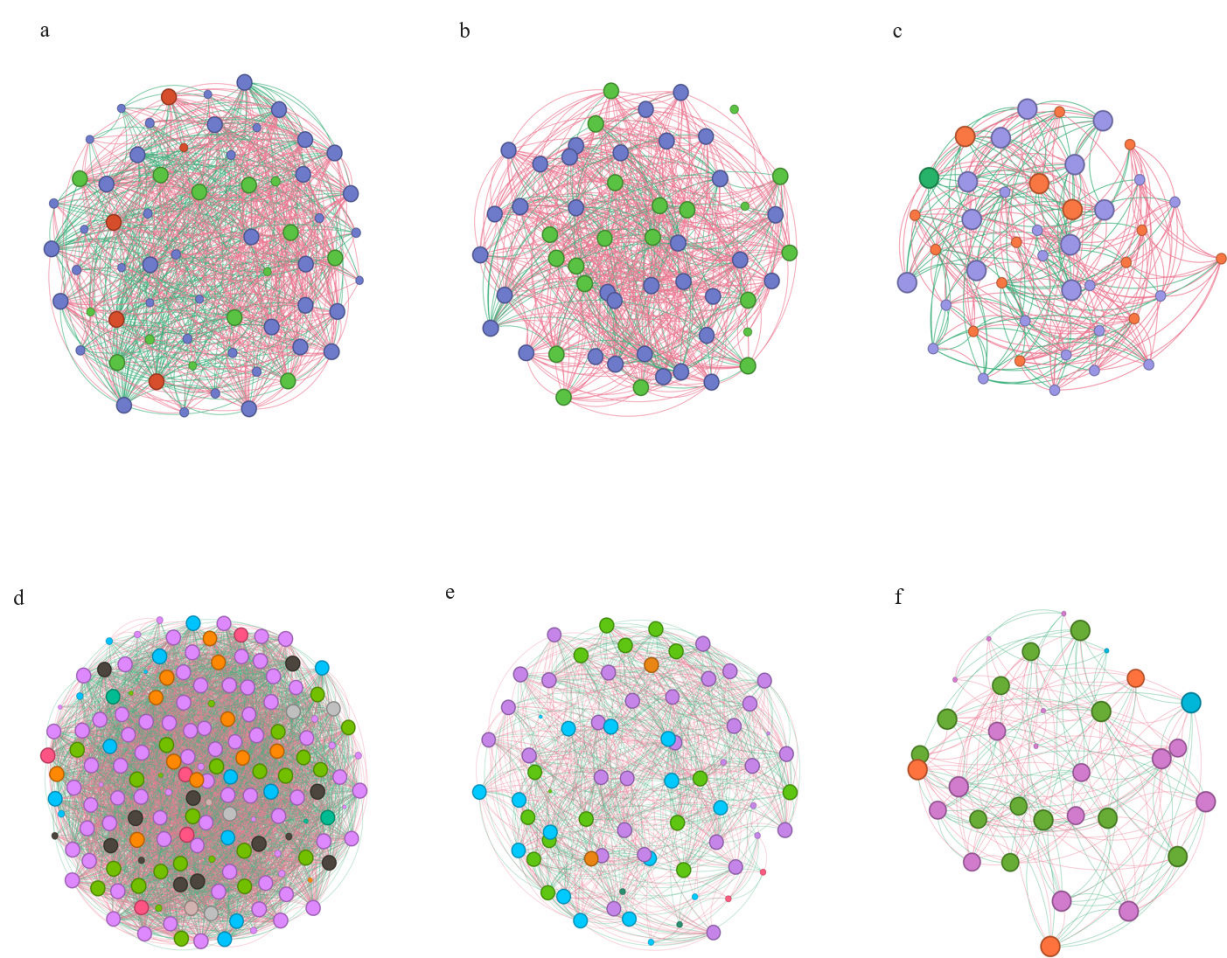
Co-occurrence networks under different seasons. Note: Co-occurrence networks of endophytic fungal in spring (a), co-occurrence networks of endophytic fungal in summer (b), co-occurrence networks of endophytic fungal in autumn (c), co-occurrence networks of endophytic bacterial in spring (d), co-occurrence networks of endophytic bacterial in summer (e), and co-occurrence networks of endophytic bacterial in autumn (f).

To compare how different seasons affect the complexity of endophytic fungi and bacteria of the root co-occurrence networks, six networks were constructed across the seasonal succession. Co-occurrence network analysis revealed that endophytic fungal and bacterial co-occurrence network connectivity and complexity decreased gradually with seasonal variation (Fig. 5). The endophytic fungi and bacteria of the root co-occurrence networks under different seasons followed different changing trajectories based on the network topological parameters. In addition, the network topology parameters also change significantly with the seasons. A total number of nodes (network size) strongly decreased with the succession of seasons, as did the total number of links and average degree (average links per node). Relative modularity (RM) is highest in autumn. Map density is highest in summer (Table 2).

**TABLE 2:** Topological properties of microbial networks

Network topology parameter	Endophytic fungi			Endophytic bacteria		
	Ar	Br	Cr	Ar	Br	Cr
Total number of nodes	63	54	43	162	71	34
Total number of links	759	628	287	4570	880	190
Positive edges (>0.7 with *P* < 0.05)	474	471	176	2420	464	113
Negative edges (<−0.7 with *P* < 0.05)	285	157	111	2150	416	77
RM (relative modularity)	0.418	0.504	0.665	0.543	0.541	0.59
Average degree (average links per node)	24.059	23.259	13.349	56.42	24.789	11.176
Map density	0.389	0.439	0.318	0.35	0.354	0.339

### Correlation analysis between the endophyte and active ingredients of *R. palmatum*

**Fig 6:**
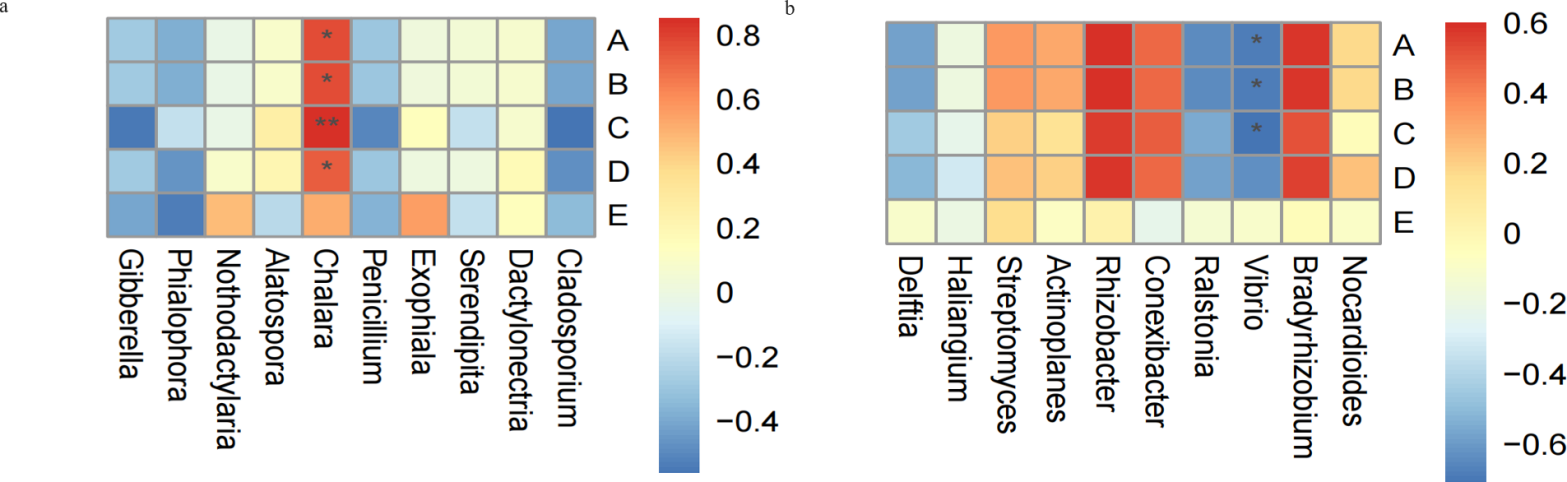
Correlation analysis between metabolites and the top ten dominant genera of endophytic fungi (a), Correlation analysis between metabolites and the top ten dominant genera of endophytic bacteria (b). Note: A is aloe-emodin, B is rhein, C is emodin, D is chrysophanol, and E is physcion. “*”indicates the differences are significant at *P* < 0.05 and “**”indicates the differences are significant at *P* < 0.01.

Correlation analysis was performed based on the relative abundance of 10 dominant fungal and bacterial genera with the active ingredients of *R. palmatum* under different seasons. Correlation analysis showed that the contents of aloe-emodin, rhein, emodin, and chrysophanol were significantly positively correlated with the dominant endophytic fungal genus *Chalara* (Fig. 6a), while the contents of aloe-emodin, rhein, and emodin were significantly negatively correlated with the dominant endophytic genus bacterial *Vibrio* (Fig. 6b).

### Function prediction of PICRUSt and FUNGuild

**Fig 7:**
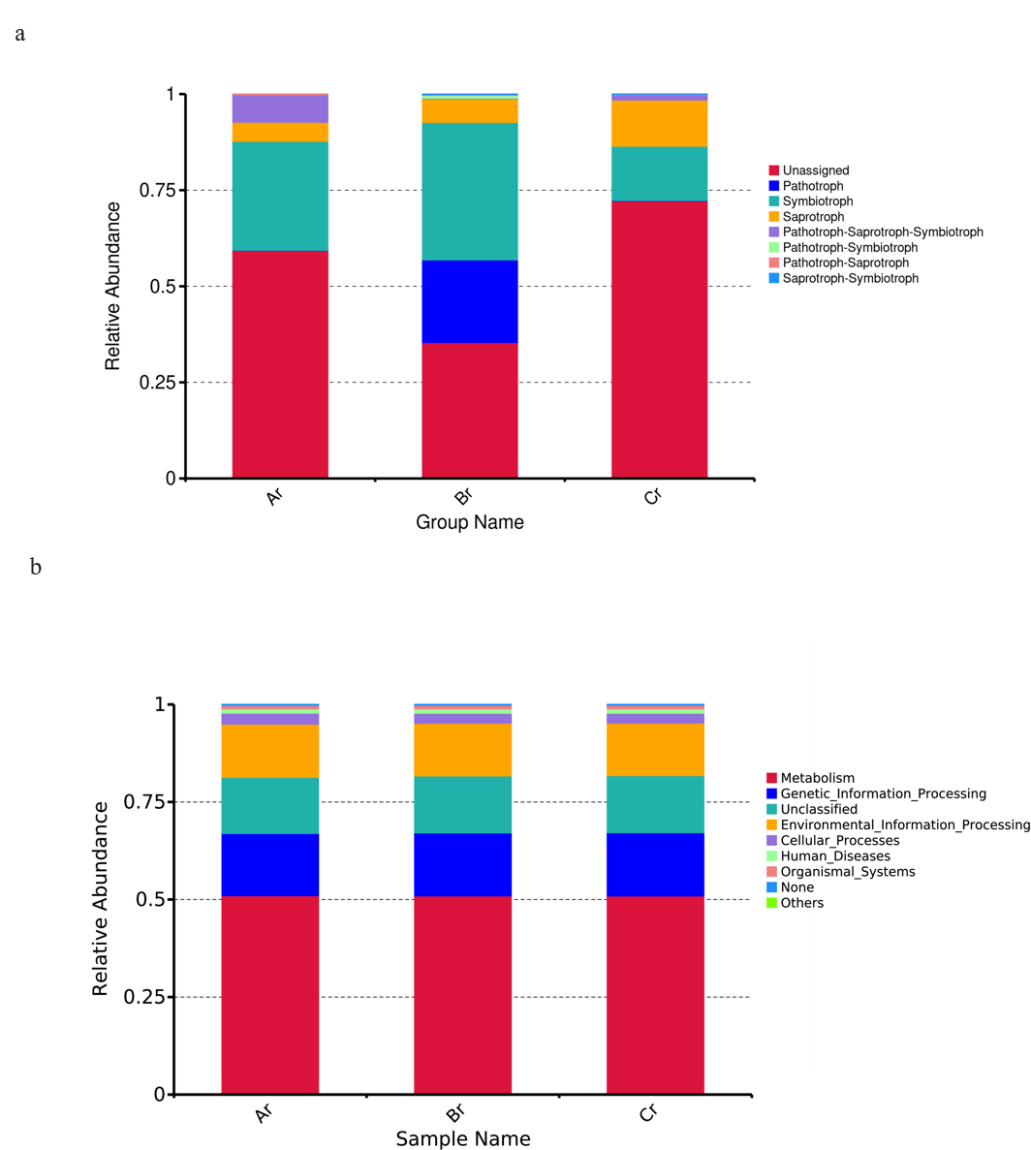
Relative abundance of predicted trophic mode of fungi (a) and relative abundance of predicted KEGG Orthologs functional profiles (KEGG level 1) of bacteria (b).

FUNGuild was used to predict the nutritional and functional groups of the fungal communities with different samples. The prediction results of endophytes functional fungi relative abundance of *R. palmatum* based on FUNGuild database showed that seven trophic mode groups could be classified, including Pathotroph, Symbiotroph, Saprotroph, Pathotroph-Saprotroph–Symbiotroph, Pathotroph–Symbiotroph, Pathotroph–Saprotroph, and Saprotroph–Symbiotroph. The trophic mode of all samples was mainly symbiotroph, and their relative abundance first increased and then decreased with the change of season (Fig. 7a).

To study bacterial function, we used PICRUSt to perform bacterial function prediction analysis. Through comparisons with the Kyoto Encyclopedia of Genes and Genomes (KEGG) database, six categories of biological metabolic pathways (primary functional level) were obtained, which included Metabolism, Genetic_Information_Processing, Environmental_Information_Processing, Cellular_Processes, Human_Diseases, and Organismal_Systems. The metabolism pathway was the primary component in all samples, accounting for 50.95%–51.04% (Fig. 7b).

## DISCUSSION

In this study, the results of alpha diversity showed that the Shannon index and Chao1 index of *R. palmatum* were different under different seasons. On the seasonal scale, the abundance and diversity of the bacterial community decreased gradually with the seasonal changes of *R. palmatum*, while the abundance and diversity of the fungal community increased first and then decreased. The results of community composition showed that the dominant fungal and bacterial phyla were Ascomycota and Proteobacteria, respectively. The relative abundance of dominant fungal phylum decreased with the change of seasons, while the relative abundance of dominant bacterial phylum increased with the change of seasons. Many studies also have shown that Ascomycota and Proteobacteria were the dominant phyla of fungal and bacterial endophytes in many plants (31–33). Both Proteobacteria and Ascomycota contain many functional strains related to denitrification and carbon and nitrogen cycling (34, 35), which indicates that the endophyte community of *R. palmatum* is mainly composed of beneficial microorganisms. At the genus level, the dominant genera of endophytes and their relative richness were different, which may be caused by differences in sunshine duration, average annual precipitation, and average annual temperature under different seasons (36, 37). In order to investigate the effects of seasonal changes on the differences between endophytic fungal and bacterial communities in *R. palmatum*, a co-occurrence network of microbial communities was constructed, and we found that endophytic fungal and bacterial co-occurrence network connectivity and complexity decreased gradually with seasonal variation.

To explore the correlation between endophytes and the active ingredients of *R. palmatum*, correlation analysis was performed based on the relative abundance of 10 dominant fungal and bacterial genera with the active ingredients of *R. palmatum* under different seasons. We found that active ingredients were significantly positively correlated with the dominant endophytic fungal genera (*Chalara*) and negatively correlated with the dominant endophytic bacterial genera (*Vibrio*)*,* which was consistent with the results of previous studies. Chen et al. showed that active ingredients of *R. palmatum* were positively correlated with the diversity and abundance of endophytic fungi (20). Plant-related bioactive substances and plant-based medicines have been a part of traditional health care in most parts of the world. In the past, research on medicinal plants has mainly focused on their composition and pharmacological effects. However, with the rapid development of molecular technology, the microbiome composition and function of medicinal plants have received more and more attention. A large number of studies believe that the microbiome directly or indirectly affects the synthesis of pharmacologically active ingredients, which provides a new idea for a sustainable production of drug resources (38, 39).

FUNGuild is often applied to compare functions and to identify specific functional classifications in the study of fungi; it also can be used in research about fungal community (40). In this study, we found that the trophic mode of all samples was mainly symbiotroph, and their relative abundance increased first and then decreased with the change of season through function prediction of FUNGuild, which indicated that seasons had a great influence on the functional fungal groups of endophytes in *R. palmatum*. Although FUNGuild has been employed to analyze the function of fungi to a certain extent, the approach has certain limitations as it is based on existing literature and data. Hence, to study the function of endophytic fungi comprehensively, we ought to further investigate the classification and functional groups of soil fungi. PICRUSt analysis is capable of predicting the metabolic function of bacterial communities with high reliability, and it has been employed to investigate bacterial functions in numerous plants (41). We used high-throughput sequencing results for PICRUSt function prediction analysis, and the results show that metabolism was the major component in all samples. This result is similar to that obtained in a study of the function of the water-level fluctuation zone of the Danjiangkou reservoir in China during the dry period of rhizosphere bacteria by Chen et al. (42). Pepe-Ranney et al. (43) found that endophytes originate from the rhizosphere microbiome, resulting in similar outcomes. Due to the limitations of PICRUSt functional prediction analysis, we were unable to reveal how endophytes affect plant metabolism through functional prediction and so on. Although the functions of certain endophytic bacterial taxa remain unclear, they demonstrate significant potential and merit further investigation. In our works, the function of the relevant endophytic bacteria was only preliminarily predicted. In the next studies, further validation should be conducted using other methods such as metagenomics, which can help to better understand the endophytic bacterial function.

### Conclusion

Our data suggest that endophytes diversity and composition of *R. palmatum* existed in seasonal dynamics, and the connectivity and complexity of endophyte co-occurrence network decreased with the change of seasons. The active ingredients of *R. palmatum* were significantly positively correlated with genera of endophytic fungi (*Chalara*)*,* which may play an important role in the accumulation of active ingredients. Our results may lay a foundation for understanding the interaction mechanism of endophyte and medicinal plant, and can also provide a theoretical basis for sustainable production of medicinal plants.

## Data Availability

All raw sequencing data have been submitted to the NCBI Sequence Read Archive (SRA) database (https://www.ncbi.nlm.nih.gov/sra) under the accession number (PRJNA1105668).
